# Supplementary material for: The mean reticulocyte volume is a valuable index in early diagnosis of cancer-related anemia
Source: PeerJ. 2024 Feb 29;12:e17063. doi: 10.7717/peerj.17063 (PMC10909343; doi:10.7717/peerj.17063)
Supplement: Supplemental Information 2 [file peerj-12-17063-s002.docx]

STROBE Statement—checklist of items that should be included in reports of observational studies

|  | Item No. | Recommendation | Page  No. | Relevant text from manuscript |
| --- | --- | --- | --- | --- |
| **Title and abstract** | 1 | (*a*) Indicate the study’s design with a commonly used term in the title or the abstract | 2 | This prospective study aimed to ....... |
|  |  | (*b*) Provide in the abstract an informative and balanced summary of what was done and what was found | 2 | Included in Methods and Results section. |
| Introduction | | | |  |
| Background/rationale | 2 | Explain the scientific background and rationale for the investigation being reported | 3-4 | 1-3 paragraphs in Introduction section. |
| Objectives | 3 | State specific objectives, including any prespecified hypotheses | 3-4 | 1. **In first paragraph:** Therefore, early identification and diagnosis of CRA will be of importance to improve the prognosis of cancer patients. 2. **In second paragraph;** Therefore, the abnormalities of some indicators reflecting the abovementioned three ways of the etiology of CRA would be closely linked with anemia, and can be used in prediction, early diagnosis, and disease assessment of CRA. 3. **In third paragraph:**   Thus, the measurements of non-tradictional reticulocyte indices will be of notable significance in the early diagnosis of CRA. However, rare reports were found about the utility of reticulocyte volume in CRA. This study was undertaken to study the utility of mean reticulocyte volume (MRV) obtained from Mindray BC-7500 blood analyzer in early identifying anemia from non-anemia in overall patients with variety of malignancies. |
| Methods | | | |  |
| Study design | 4 | Present key elements of study design early in the paper | 5 | **Study population:**  This ia a prospective study. |
| Setting | 5 | Describe the setting, locations, and relevant dates, including periods of recruitment, exposure, follow-up, and data collection | 5 | **Study population:**  The subjects were recruited from several departments in the Cancer Center of Zhejiang Provincial People's Hospital between August 2022 and May 2023, including 136 males and 148 females, aged 37-85 years. They included 114 non-anemic and 170 anemic cancer patients. The distribution of different type of cancers was listed in **Table 1**. |
| Participants | 6 | (*a*) *Cohort study*—Give the eligibility criteria, and the sources and methods of selection of participants. Describe methods of follow-up  *Case-control study*—Give the eligibility criteria, and the sources and methods of case ascertainment and control selection. Give the rationale for the choice of cases and controls  *Cross-sectional study*—Give the eligibility criteria, and the sources and methods of selection of participants | 5 | **Study population:**  The diagnosis of anemia was made according to the criteria:.........7) presence of acute infection. |
|  |  | (*b*) *Cohort study*—For matched studies, give matching criteria and number of exposed and unexposed  *Case-control study*—For matched studies, give matching criteria and the number of controls per case |  |  |
| Variables | 7 | Clearly define all outcomes, exposures, predictors, potential confounders, and effect modifiers. Give diagnostic criteria, if applicable | 5-7 | 1. **Study population:**   The diagnosis of anemia was made according to the criteria:   1. Included in **Laboratory assays** section. |
| Data sources/ measurement | 8* | For each variable of interest, give sources of data and details of methods of assessment (measurement). Describe comparability of assessment methods if there is more than one group | 5-6 | 1. Included in **Laboratory assays** section. |
| Bias | 9 | Describe any efforts to address potential sources of bias | 6 | **Statistical analysis** section:  Initially, the Kolmogorov–Smirnov test was used to ..........Mann–Whitney *U* test and Student’s *t*-test, respectively. |
| Study size | 10 | Explain how the study size was arrived at. | 4-5 | **In Sample size** section. |

Continued on next page

| Quantitative variables | 11 | Explain how quantitative variables were handled in the analyses. If applicable, describe which groupings were chosen and why | 6 | **Statistical analysis** section:  Initially, the Kolmogorov–Smirnov test was used to analyze the normality of the data distribution, and they are presented as the mean ± standard deviation ($\bar{x}\pm sd$) and median when distributing normally and non-normally, respectively. The non-normal and normal distribution data of the patient’s characteristics were analyzed by Mann–Whitney *U* test and Student’s *t*-test, respectively. |
| --- | --- | --- | --- | --- |
| Statistical methods | 12 | (*a*) Describe all statistical methods, including those used to control for confounding | 6 | Included i**n Statistical analysis** section: |
|  |  | (*b*) Describe any methods used to examine subgroups and interactions | 6 | Included i**n Statistical analysis** section: |
|  |  | (*c*) Explain how missing data were addressed | 6 | **Statistical analysis** section:  In this study, the included subjects were far more than that of the minimal sample size (including the 20% possible missing samples), thus some missing data did not influence the statistics. |
|  |  | (*d*) *Cohort study*—If applicable, explain how loss to follow-up was addressed  *Case-control study*—If applicable, explain how matching of cases and controls was addressed  *Cross-sectional study*—If applicable, describe analytical methods taking account of sampling strategy |  | Not applicable. |
|  |  | (*e*) Describe any sensitivity analyses | 6 | The receiver operating characteristic (ROC) curves were established to calculate the area under the curve (AUC), and the identifying ability (sensitivity and specificity）of MRV between non-anemia and anemia in cancer patients was evaluated |
| Results | | | | |
| Participants | 13* | (a) Report numbers of individuals at each stage of study—eg numbers potentially eligible, examined for eligibility, confirmed eligible, included in the study, completing follow-up, and analysed | 7 | **Results**  ***Basic physiological and laboratory characteristics of cancer patients:***  In this study, 284 consecutive patients........ A total of 23 and 21 types of tumors were included ......, the total percentage of the main malignancies........most frequently ...... no statistical difference between anemia and non-anemia patients (60/176, 34% vs. 31/108, 28.7%, p>0.05) (**Table 1**). The mean age of non-anemia ......(54.5±15.9 vs. 62.1±15.7, p<0.001), and male and female ...........(males: 49% vs. 54% , p>0.05). |
|  |  | (b) Give reasons for non-participation at each stage |  | Not applicable. |
|  |  | (c) Consider use of a flow diagram |  | Not applicable. |
| Descriptive data | 14* | (a) Give characteristics of study participants (eg demographic, clinical, social) and information on exposures and potential confounders | 7-8 | **Results**  **Basic physiological and laboratory characteristics of cancer patients:**  Table 1, Table 2.  **Adjusted multivariate analysis of MRV and Hb for risk of CRA:**  Based on ...... some confounders such as gender, age, BMI, SBP, and DBP that could potentially affect the production and release of reticulocyte were included to perform an adjusted multivariate regression analysis. |
|  |  | (b) Indicate number of participants with missing data for each variable of interest | 7 | The detailed comparisons of the basic biological and laboratory characteristics are presented **in **Table** 2.** |
|  |  | (c) *Cohort study*—Summarise follow-up time (eg, average and total amount) |  |  |
| Outcome data | 15* | *Cohort study*—Report numbers of outcome events or summary measures over time |  |  |
|  |  | *Case-control study—*Report numbers in each exposure category, or summary measures of exposure |  |  |
|  |  | *Cross-sectional study—*Report numbers of outcome events or summary measures | *7* | **Results**  **Basic physiological and laboratory characteristics of cancer patients:**  The study initially investigated ..... with anemia and non-anemia, respectively. A total of 23 and 21 types of tumors were included in anemia and non-anemia group, respectively. ......the total percentage of the main malignancies......showed no statistical difference between anemia and non-anemia patients ..... (**Table 1**). The mean age of non-anemia patients......than that of anemia one........in groups non-anemia and anemia (males: 49% vs. 54% , p>0.05). There was no .... between the two groups (p>0.05), ...... presented **in **Table** 2.** |
| Main results | 16 | (*a*) Give unadjusted estimates and, if applicable, confounder-adjusted estimates and their precision (eg, 95% confidence interval). Make clear which confounders were adjusted for and why they were included | 8 | **Results**  **Adjusted multivariate analysis of MRV and Hb for risk of CRA:**  Based on the anemia classified...., some confounders such as gender, age, BMI, SBP, and DBP that could potentially affect ... .to perform an adjusted multivariate regression analysis. ..... the risk of anemia increased by almost 0.1 and 0.04 times for each 1 fl and 1g reduction of MRV and Hb levels, respectively. When MRV and Hb were treated as...... from ROC curve analyse, the overall risk.....was 19 and 4 times higher than that ....( **Table 6**). |
|  |  | (*b*) Report category boundaries when continuous variables were categorized | 8 | When MRV and Hb were treated as categorical variables expressed as “increased” or “decreased” by using the optimal cutoff values of male and female patients from ROC curve analyse, the overall risk of anemia in patients with below the cutoff values was 19 and 4 times higher than that with above the cutoff values, respectively |
|  |  | (*c*) If relevant, consider translating estimates of relative risk into absolute risk for a meaningful time period |  | Not applicable. |

Continued on next page

| Other analyses | 17 | Report other analyses done—eg analyses of subgroups and interactions, and sensitivity analyses | 7-8 | **Results**  **1.Associations of MRV levels with the RBC and reticulocyte indices** section  **2.ROC curves analyse of MRV in identifying CRA diagnosed by RHE and RPI** section  **3.RBC and reticulocyte indices levels in anemia and non-anemia patients classified by MCHC, MCH and MCV** section |
| --- | --- | --- | --- | --- |
| Discussion | | | | |
| Key results | 18 | Summarise key results with reference to study objectives | 9-11 | In **Discussion** section. |
| Limitations | 19 | Discuss limitations of the study, taking into account sources of potential bias or imprecision. Discuss both direction and magnitude of any potential bias | 11-12 | Within our knowledge, there is a limitation in this study...... |
| Interpretation | 20 | Give a cautious overall interpretation of results considering objectives, limitations, multiplicity of analyses, results from similar studies, and other relevant evidence | 12 | Despite the limitation, our study also revealed that decreased MRV level on Mindray BC-7500 analyzer were strongly correlated with early anemia in cancer patients.  **Conclusion**  This study revealed that the MRV on Mindray BC-7500 canbe used as a sensitive predictor in early diagnosis of cancer-related anemia, and decreased MRV level may be the powerful risk factor of overt anemia in cancer patients. However, further studies from multi-center and different analyzers with more diverse patients with cancer-related anemia are needed to draw more definitive conclusions. |
| Generalisability | 21 | Discuss the generalisability (external validity) of the study results | 11 | As a convenient and fast calculated index on Mindray BC-7500 analyzer, MRV demonstrated its special clinical value in the diagnosis and risk evaluation of CRA. In recent years, few studies have evaluated the significance of MRV in CRA because it is an unavailable index on most analyzers and is not commonly used to predict and assess risk in overt CRA. Similarly, the present study suggests that the MRV is an independent risk factor for CRA. |
| Other information | |  | | |
| Funding | 22 | Give the source of funding and the role of the funders for the present study and, if applicable, for the original study on which the present article is based | 12 | In **Funding Statement** section. |

*Give information separately for cases and controls in case-control studies and, if applicable, for exposed and unexposed groups in cohort and cross-sectional studies.

**Note:** An Explanation and Elaboration article discusses each checklist item and gives methodological background and published examples of transparent reporting. The STROBE checklist is best used in conjunction with this article (freely available on the Web sites of PLoS Medicine at http://www.plosmedicine.org/, Annals of Internal Medicine at http://www.annals.org/, and Epidemiology at http://www.epidem.com/). Information on the STROBE Initiative is available at www.strobe-statement.org.
